# Association Between K–12 School Mask Policies and School-Associated COVID-19 Outbreaks — Maricopa and Pima Counties, Arizona, July–August 2021

**DOI:** 10.15585/mmwr.mm7039e1

**Published:** 2021-10-01

**Authors:** Megan Jehn, J. Mac McCullough, Ariella P. Dale, Matthew Gue, Brian Eller, Theresa Cullen, Sarah E. Scott

**Affiliations:** ^1^School of Human Evolution and Social Change, Arizona State University, Tempe, Arizona; ^2^College of Health Solutions, Arizona State University, Phoenix, Arizona; ^3^Epidemic Intelligence Service, CDC; ^4^Disease Control Division, Maricopa County Department of Public Health, Phoenix, Arizona; ^5^Pima County Health Department, Tucson, Arizona.

CDC recommends universal indoor masking by students, staff members, faculty, and visitors in kindergarten through grade 12 (K–12) schools, regardless of vaccination status, to reduce transmission of SARS-CoV-2, the virus that causes COVID-19 (*1*). Schools in Maricopa and Pima Counties, which account for >75% of Arizona’s population (*2*), resumed in-person learning for the 2021–22 academic year during late July through early August 2021. In mid-July, county-wide 7-day case rates were 161 and 105 per 100,000 persons in Maricopa and Pima Counties, respectively, and 47.6% of Maricopa County residents and 59.2% of Pima County residents had received at least 1 dose of a COVID-19 vaccine. School districts in both counties implemented variable mask policies at the start of the 2021–22 academic year (Table). The association between school mask policies and school-associated COVID-19 outbreaks in K–12 public noncharter schools open for in-person learning in Maricopa and Pima Counties during July 15–August 31, 2021, was evaluated.

**TABLE:** School-associated COVID-19 outbreaks and school characteristics among K–12 public noncharter schools, by school mask policy — Maricopa and Pima Counties, Arizona, July–August 2021

Characteristic	All schools no. (%)	School mask requirements no. of schools (%)				p-value*
		None*	Early*		Late*	
	(N = 999)	(n = 480)	(n = 210)		(n = 309)	
**School-associated outbreak^†^**						<0.001
No	808 (81)	367 (76)	194 (92)		247 (80)	
Yes	191 (19)	113 (24)	16 (8)		62 (20)	
**County**						<0.001
Maricopa	782 (78)	444 (93)	100 (48)		238 (77)	
Pima	217 (22)	36 (8)	110 (52)		71 (23)	
**Grades present^§^**						NC^§^
Elementary (K–5)	678 (68)	296 (62)	136 (65)		246 (80)	
Middle (6–8)	656 (66)	336 (70)	110 (52)		210 (68)	
High (9–12)	251 (25)	160 (33)	58 (28)		33 (11)	
**7-day case rate in school zip code** ^¶^						0.002
<10	3 (0.3)	3 (0.6)	0 (—)	0 (—)		
10 to <50	4 (0.4)	4 (0.8)	0 (—)	0 (—)		
50 to <100	36 (4)	14 (3)	19 (9)	3 (1)		
>100	956 (96)	459 (96)	191 (91)	306 (99)		
Title I status**						<0.001
Not Title I	359 (36)	216 (45)	45 (21)	98 (32)		
Title I eligible	81 (8)	48 (10)	5 (2)	28 (9)		
Any Title I participation	559 (56)	216 (45)	160 (76)	183 (59)		
**No. of students enrolled**						<0.001
<850	243 (24)	60 (13)	109 (52)	74 (24)		
850–1,199	248 (25)	108 (23)	32 (15)	108 (35)		
1,200–1,649	255 (26)	156 (33)	32 (15)	67 (22)		
≥1,650	253 (25)	156 (33)	37 (18)	60 (19)		

**Abbreviations:** K–12 = kindergarten through grade 12; NC = not calculated.

* Chi-square and Fisher’s exact tests were used to calculate p-values between schools with early mask requirements (mask requirement in place at the start of the school year) and those with no mask requirements, which are included in logistic regression analyses. Schools with late mask requirements instituted mask requirements at any time after the start of the school year.

^†^ During July 15–August 31, 2021.

^§^ Defined as the presence or absence of grades taught at the school. Categories are not mutually exclusive, and p-value was not calculated. Three separate indicator variables were used to capture presence of these grade levels in the multivariate model.

^¶^ Calculated as all new confirmed and probable COVID-19 cases per 100,000 population occurring in each zip code containing a school included in this analysis during the surveillance week in which the school’s academic year started. Categories presented are based on CDC community transmission metrics, included as a continuous variable in the multivariate model.

** Under Title I, financial assistance is provided to local educational agencies and schools with high numbers or high percentages of students from low-income families.

A school was considered to have a mask requirement if all persons, regardless of vaccination status, were required to wear a mask indoors in school. An early mask requirement was one that was in place when the school year began, and a late mask requirement was one that was implemented any time after school began. Mask policies were abstracted from publicly available school COVID-19 mitigation plans, which must be posted online per Executive Order 2020–51.^†^ A school-associated outbreak was defined as the occurrence of two or more laboratory-confirmed COVID-19 cases^§^ among students or staff members at the school within a 14-day period and at least 7 calendar days after school started, and that was otherwise consistent with the Council for State and Territorial Epidemiologists 2020 outbreak definition^¶^ and Arizona’s school-associated outbreak definition.** In Arizona, school-associated outbreaks are required to be reported to the local public health agency within 24 hours; data are stored in Arizona’s Medical Electronic Disease Surveillance Intelligence System. School characteristics, including county of location, grade levels present,^††^ enrollment, and Title I status^§§^ (a measure of a school population’s socioeconomic status) were obtained from the Arizona Department of Education. Crude and adjusted logistic regression analyses with 95% confidence intervals (CIs) were performed in Stata (version 15; StataCorp) and adjusted for school county, enrollment size, grade levels present, Title I status, and 7-day COVID-19 case rate in the school’s zip code during the week school commenced. Schools with late mask requirements were excluded from these analyses because of their mixed exposure status during the sampling time frame (e.g., schools might have enacted mask requirements after an outbreak). Vaccination coverage for staff members and students was not available at the school level.

Data were available for 1,020 of 1,041 (98.0%) K–12 public noncharter schools in Maricopa and Pima counties. Twenty-one (2.0%) schools had outbreaks reported <7 days after school began and were excluded from the analyses. Among the 999 (96.0%) schools included in the analysis, 210 (21.0%) had an early mask requirement, 309 (30.9%) had a late mask requirement enacted a median of 15 days after school started (interquartile range = 9–17 days), and 480 (48.0%) had no mask requirement (Table). During July 15–August 31, 2021, 191 school-associated outbreaks occurred, 16 (8.4%) in schools with early mask requirements, 62 (32.5%) in schools with late mask requirements, and 113 (59.2%) in schools without a mask requirement.

In the crude analysis, the odds of a school-associated COVID-19 outbreak in schools with no mask requirement were 3.7 times higher than those in schools with an early mask requirement (odds ratio [OR] = 3.7; 95% CI = 2.2–6.5). After adjusting for potential described confounders, the odds of a school-associated COVID-19 outbreak in schools without a mask requirement were 3.5 times higher than those in schools with an early mask requirement (OR = 3.5; 95% CI = 1.8–6.9).

CDC recommends universal indoor masking in K–12 schools (*1*); however, masking requirements in K–12 schools vary by school district, county, and state. In the two largest Arizona counties, with variable K–12 school masking policies at the onset of the 2021–22 academic year, the odds of a school-associated COVID-19 outbreak were 3.5 times higher in schools with no mask requirement than in those with a mask requirement implemented at the time school started. Lapses in universal masking contribute to COVID-19 outbreaks in school settings (*3*); CDC K–12 school guidance recommends multiple prevention strategies. Given the high transmissibility of the SARS-CoV-2 B.1.617.2 (Delta) variant, universal masking, in addition to vaccination of all eligible students, staff members, and faculty and implementation of other prevention measures, remains essential to COVID-19 prevention in K–12 settings (*1*).

## Footnotes

†
https://azgovernor.gov/sites/default/files/executive_order_2020-51.pdf

§ Defined as a SARS-CoV-2–positive reverse transcription–polymerase chain reaction or nucleic acid amplification test or antigen test.

¶
https://preparedness.cste.org/wp-content/uploads/2020/08/Educational-Outbreak-Definition.pdf

** Emergency Measure 2020–03. https://www.azdhs.gov/covid19/documents/emergency-measure-2020-03.pdf

†† The variable for grade levels present was included within the model as three separate indicator variables, corresponding to elementary, middle, and high school.

§§
https://www2.ed.gov/programs/titleiparta/index.html
